# Quality of Life Impact of Hypoglossal Nerve Stimulation with Inspire^®^ Device in Patients with Obstructive Sleep Apnea Intolerant to Continuous Positive Airway Pressure Therapy

**DOI:** 10.3390/life12111737

**Published:** 2022-10-28

**Authors:** Peter Baptista, I. Madeleine Di Frisco, Elena Urrestarazu, Juan Alcalde, Manuel Alegre, Isabel Sanchez, Carlos O’Connor-Reina, Guillermo Plaza

**Affiliations:** 1 Otorhinolaryngology Department, University Clinic of Navarra, 31007 Pamplona, Spain; 2 Pulmonology Department, Clínica Universidad de Navarra, 31007 Pamplona, Spain; 3 Otorhinolaryngology Department, Hospital Quironsalud Marbella, 29603 Malaga, Spain; 4 Otorhinolaryngology Department, University Hospital of Fuenlabrada, University Rey Juan Carlos, 28042 Madrid, Spain

**Keywords:** quality of life, obstructive sleep apnea, hypoglossal nerve stimulation, minimally invasive surgical technique, EuroQol-5D-5L

## Abstract

Patients with obstructive sleep apnea (OSA) that do not tolerate/accept continuous positive airway pressure (CPAP) are candidates for surgical alternatives. Hypoglossal nerve stimulation (HNS) through the implantation of the Inspire^®^ device constitutes a minimally invasive operative option. The main objective of this study is to estimate, under real-world clinical practice conditions, the 3-month impact on the quality of life (IQoL) of the HNS in patients with moderate/severe OSA who do not tolerate or accept CPAP, compared to patients who did not receive HNS. As a baseline, the unadjusted EuroQol utility index was 0.764 (SD:0.190) in the intervention group (IGr) and 0.733 (SD:0.205) in the control group (CGr); three months later, the indexes were 0.935 (SD: 0.101) and 0.727 (SD:0.200), respectively. The positive impact on quality of life was estimated to be +0.177 (95% CI: 0.044–0.310; *p* = 0.010). All dimensions in the IGr improved compared to CGr, especially for usual activities (*p* < 0.001) and anxiety/depression (*p* > 0.001). At the end of the follow-up, there was no significant difference in the quality of life between the general Spanish population and the IGr (difference: 0.012; CI95%: −0.03 to −0.057; *p* = 0.0578) for the same age range; however, there was a difference concerning the CGr (difference: −0.196; CI95%: −0.257 to −0.135; *p* < 0.001). In conclusion, patients with moderate/severe OSA implanted with the Inspire^®^ device showed a positive IQoL.

## 1. Introduction

Obstructive sleep apnea (OSA) is defined by the presence of an apnea-hypopnea index (AHI) ≥ 5/h accompanied by excessive daytime sleepiness, unrefreshing sleep, extreme tiredness, and/or impaired quality of life (QoL) related to sleeping or not justified by other causes [1]. OSA increases the risk of incidence of cardiovascular disease [2], and traffic accidents [3], as well as reduced QoL [4]. A prevalence of 26.2% in men and 28% in women (age 30–70 years) is estimated [5].

The initial recommended treatment consists of continuous positive airway pressure (CPAP) in patients with excessive daytime sleepiness (Epworth sleepiness scale (ESS) > 10), and QoL alterations related to sleeping and/or arterial hypertension [6]. CPAP delivers positively pressurized air through an interface that fits over the nose or nose and mouth while the patient sleeps. It has shown high efficacy in keeping the upper respiratory tract patent, avoiding episodes of apnea; however, its use is not exempt from problems for the patient, such as the noise it generates—which can limit night rest—skin, dental, and bone (mandibular) discomfort. It has been calculated that 14.9–35.5% of patients do not tolerate CPAP [7,8]. Non-adherence to CPAP is defined as the use of fewer than 4 h per night on 70% of nights. This circumstance may require the use of alternative therapy, involving the use of invasive surgical techniques such as uvulopalatopharyngoplasty or tongue base surgery, and maxillomandibular advancement.

In recent years, hypoglossal nerve stimulation (HNS) has been shown to be a minimally invasive technique that acts through synchronized stimulation of the upper airways, improving the severity of OSA in patients [9].

Inspire^®^ is a type of HNS that is synchronized with breathing [10]. It produces a contraction of the genioglossus and geniohyoid muscles of the tongue with repercussions on the palate through the palatoglossus muscle [11], facilitating the patency of the upper respiratory tract. It consists of a pulse generator implanted in the right ipsilateral mid-infraclavicular region, like a pacemaker. The pulse generator is connected to a sensing lead located in the internal and external intercostal muscles to detect ventilatory effort. It is also connected to a stimulation electrode, placed over the protruding fibers of the right hypoglossal. An external remote control is used to turn on the device. Inspire^®^ is indicated in patients >18 years old, with a BMI <35, moderate/severe OSA, AHI >15, and inadequate adherence/tolerance or rejection of CPAP. Its safety and efficacy were shown in the STAR trial, estimating a significant reduction compared to the control group, of the AHI at one week (difference: −16.9; 95% CI: −24.7 to −9; *p* < 0.001) [12] and at 12 weeks (difference: −30.4 ± 10.4 to −13.5 ± 14.3) as well as at 36 months (−11.5 ± 13.9) [13]; furthermore, the EFFECT study [14] associated HNS with a reduction in the severity of moderate/severe OSA (AHI difference: −15.5; 95% CI: −18.3 to −12.8) and sleepiness (ESS difference: −3.3; 95% CI: −4.4 to −2.2), as well as an improvement in QoL.

Although shown to be safe and effective in clinical practice [15,16,17,18,19], our health system does not yet finance the device, and the patient must bear the cost of the device.

The main objective of this study is to estimate the impact on quality of life (IQoL) in real-life conditions and after a 3-month follow-up in patients with moderate/severe OSA that were implanted with the Inspire device compared to the group of patients who did not receive HNS. A secondary objective was to compare the QoL of implanted patients with those of the Spanish general population.

## 2. Materials and Methods

### 2.1. Study Design and Population

An observational study with a 3-month follow-up was conducted retrospectively in patients diagnosed with OSA who did not tolerate or did not accept standard CPAP treatment. The Navarra Drug Research Ethics Committee approved this study (number: PI_2021/129).

The study population was comprised of all patients with moderate/severe OSA who showed intolerance or did not accept CPAP treatment and were offered an alternative therapy with HNS through the implantation of the Inspire^®^ device.

Inclusion criteria consisted of patients older than 18 years, diagnosis of moderate–severe OSA according to a polysomnography study, with an AHI >15 events/hour, and who did not tolerate CPAP; the exclusion criteria: concentric collapse at the level of the soft palate during the drug-induced sleep endoscopy (DISE), pregnant women, patients with psychiatric disorders or with a significant component of insomnia, and/or BMI > 35.

### 2.2. Study Groups

Eligibility for the HNS was analyzed in patients who did not tolerate or did not accept CPAP. All patients signed an informed consent document and then were assigned to the study groups: the intervention group (IGr) was made up of patients in our center who accepted the Inspire^®^ implant; the control group (CGr) was comprised of those who did not accept the implant (currently, it is not financed by the public health system), in a 2:1 ratio. Patient recruitment was carried out between March 2016 and March 2021.

#### 2.2.1. Control Group

The CGr patients continued with CPAP once they rejected HNS. They were also counseled on how to improve CPAP adaptation either by changing their face mask or the use of a humidifier. Patients who did not tolerate CPAP were advised to consider a surgical treatment to optimize CPAP use through nasal surgery or to reduce their AHI through multilevel surgery (including palatopharyngoplasty and partial resection of the base of the tongue), as required.

#### 2.2.2. Intervention Group

The IGr was formed by those patients who accepted the HNS and were implanted with the Inspire^®^ device. Patients were discharged within less than 24 h post-intervention. Chest and submandibular stitches were removed after 10 days. Restrictions on physical activity were given for the first month post-implantation and included not lifting heavy objects and avoiding extreme right arm extension movements—such as swimming—during the first week, keeping the arm close to the body, and then allowing mild and gradual movements until full functionality was reached at 4 weeks.

One month after implantation, the patients were evaluated at the clinic, and the implant was programmed and activated. Patients were taught to operate the remote control (activate, deactivate, and pause) by themselves. At 3 months post-implant, the patients were scheduled for a polysomnography study to further adjust the device according to the patient’s needs.

### 2.3. Primary and Secondary Variables

The primary variable was the IQoL at 3 months of follow-up in the IGr in comparison with the CGr. In each group, the QoL values were estimated using the utility index obtained with the EuroQol-5D-5L questionnaire (EQ-5D-5L), before and 3 months after acceptance or not of the implant. EQ-5D-5L is a generic instrument that has already been used to measure the quality of life in patients with OSA [20,21]. It is made up of two parts. The first contains a question about each of the five dimensions that are evaluated (mobility, self-care, usual activities, pain/discomfort, and anxiety/depression), estimating the severity of each one based on five levels (from “no problem” to “impossibility” of doing something). Each severity level is coded with values between one and five, forming a five-digit sequence, which defines a single utility value of the patient’s health status; this value reflects the patient’s preference for a particular state of health. For example, result 11111 corresponds to an EQ-5D-5L utility index of 1.0, which is associated with perfect health. The second part of the instrument is a vertical visual analog scale, ranging from 0 (the worst imaginable form of health) to 100 (the best possible), where the patient indicates his or her state of health on the day it was performed; it serves as a complement to the questionnaire. Rollón-Mayordomo et al. [22] have analyzed the concept of minimal important clinical difference based on the estimate of the size effect, described by Kaziz et al. [23], indicating that, although there is no absolute formula for estimating this mentioned minimal difference, an effect size of 0.5 would be clinically relevant

Likewise, there was an inclusion of sociodemographic (sex, age), and clinical variables (body mass index (BMI), AHI, ESS, previous use of CPAP, and its use of more or less than 4 h daily) and previous presence of comorbidities (hypertension, diabetes mellitus (DM), myocardial infarction (MI), asthma, chronic obstructive pulmonary disease (COPD), chronic renal failure (CRF), dyslipidemia, cognitive failure, chronic pain, and restless legs syndrome (RLS)). QoL values were adjusted for variables showing a significant correlation.

Before the patient decided whether or not to accept the device, the EQ-5D-5L questionnaire was administered to all patients, giving an index value for the intervention (QoL_I_pre_) and control (QoL_C_pre_) groups. Subsequently, 3 months after, the intervention (QoL_I_post_) and control (QoL_C _post_) groups answered the questionnaire again.

Since the study was retrospective, it was not feasible to randomize patients; thus, the IQoL associated with the device implantation was estimated using the difference-in-differences method [24,25]. This technique applies a double difference, comparing the changes over time between both study groups and not necessarily requiring that the observed characteristics in both groups be similar, as occurs in the randomized clinical trial.

Finally, to put the quality of life of both cohorts into context, a comparison of the values QoL was made in each study group with the mean value of the Spanish population for the same age range [26,27].

### 2.4. Statistical Analysis

Continuous (mean, standard deviation) and categorical (frequency, percentage) variables were subject to descriptive statistical analysis. Using the Kolmogorov–Smirnov or Shapiro–Wilk test, the normality of the distributions of the variables was checked depending on the sample size. In addition, the Mann–Whitney or Student’s t-tests were used to compare the values of each group’s variables depending on whether the samples were normal. The difference estimator (IQoL) was calculated as:

(1) $$IQoL=\left(QoL_{I_{post}}-QoL_{C_{post}}\right)-\left(QoL_{I_{pre}}-QoL_{C_{pre}}\right)$$

This estimator was adjusted using the multivariate linear regression model:

(2) $$QoL_{iT}=\beta_{1}+\beta_{2}\times Device+\beta_{3}\times Time+\delta \times \left(Device\times Time\right)$$

where *QoL_iT_* is the *QoL* result for patient i at time *T*; the Device variable is dichotomous (0: rejects device; 1: accepts device); the Time variable is dichotomous (0: before the patient’s decision; 1: 3 months after the decision); *δ* × (*Device* × *Time*) is the interaction between both variables and *β*, each coefficient that affects each variable.

The analysis was performed using the statistical software IBM SPSS Statistics for Windows, v21.0. Armonk, NY, USA: IBM Corp. Significance was set at *p* < 0.05 for all analyses.

## 3. Results

### 3.1. Patients

Patients who presented intolerance to CPAP and met the inclusion criteria were offered the possibility of HNS with the Inspire^®^ device. In case of rejection, they continued with the existing treatment. All those who accepted joined the IGr (*n* = 22), while those who rejected it (reasons: economic: 81.8%; non-perception of the problem: 9.1%; aesthetic: 4.5%) went on to form the CGr (*n* = 44).

The baseline characteristics of the patients in the study groups are shown in Table 1, showing intergroup homogeneity. All the patients included in the study completed an individual follow-up at 3 months.

The intervention group improved their AHI from 42.9 events/h (SD: 21.0) to 14.3 events/h (SD: 7.1) (*p* < 0.0001) in the postoperative period.

### 3.2. Impact on Quality of Life

Utility index values were adjusted for ESS, DM, MI, COPD, CRF, cognitive failure, and chronic pain. Before the decision to implant the device or not, the estimate useful index of EQ-5D-5L was similar between the intervention and control groups, showing no statistically significant difference (difference: 0.013; *p* = 0.410 adjusted). At the end of follow-up, the utility index was higher, with statistical significance, in the intervention compared to the control (diff.: −0.049; *p* = 0.003 adjusted), showing an improvement in the quality of life of implanted patients (Table 2).

The regression model estimated a positive IQoL of +0.177 (95% CI: 0.044–0.310; *p* = 0.010), which implies an improvement in the quality of life of implanted patients compared to non-implanted patients. The IQoL value after adjustment was +0.062 (CI95%: 0.017–0.107; *p* = 0.007).

At the end of follow-up, the disaggregated analysis for each dimension of quality of life showed that the percentage of patients who did not present any problem in each dimension was significantly higher in the intervention group for all dimensions, mainly in daily activities (difference: 59.1%; SD: 10.6; *p* < 0.001) and anxiety/depression (difference: 54.5%; SD: 11.2; *p* < 0.001) (Table 3).

### 3.3. Quality of Life of Patients Compared to the General Spanish Population

At the end of follow-up, the mean value of the utility index for the intervention group was significantly equivalent to the mean value of the Spanish population for the same age range (0.923; SE: 0.01), while a significant reduction was observed in the control group compared to the Spanish population (Table 4).

The estimated value in all dimensions was significantly lower in the control group compared to the general population. Therefore, patients with moderate to severe OSA that do not receive treatment have a worse quality of life (Table 5).

## 4. Discussion

The study shows that HNS was associated with a positive impact on the quality of life in patients implanted with the Inspire^®^ device compared to those who did not accept the implant. The increase in QoL was observed in all the dimensions evaluated, reaching a usefulness index of the EQ-5D-5L equivalent to that estimated for the Spanish population. Those patients that rejected implantation but were ideal candidates for the implant remained with a reduced quality of life.

These results align with Kompelli et al. systematic review [28]. They analyzed the available studies with the HNS, concluding that it is a safe and effective treatment for OSA refractory to CPAP. Like those of Kent et al. [29], our results support HNS as a viable option in selected patients with moderate to severe OSA who do not tolerate or do not accept CPAP therapy, improving the symptoms and the quality of life of patients.

OSA is associated with a significant reduction in QoL compared to the general population [30], with CPAP being the standard treatment for moderate–severe intensity. Lloberes et al. [31] showed that untreated OSA patients had worse QoL than the general population but improved with CPAP treatment after 3 months. However, CPAP frequently exhibits inadequate adherence. Batool-Anwar et al. [32] showed that only a third of patients who used CPAP for <2 h per night significantly improved their QoL. A systematic review [33] showed that CPAP adherent patients, reached a significant improvement in the physical dimension of QoL, but not in the psychological dimension. Bjornsdottir et al. [4] showed that although CPAP improves QoL, patients did not always reach the levels of the general population, particularly in the physical dimension.

It is noteworthy that in our study more than three-quarters of the patients rejected the device for economic reasons. The HNS device and implant procedure is currently not included in the portfolio of health benefits of our national health system; therefore, the patient must bear the related expense themselves. This situation leads to inequity, as the improvement in the patient’s quality of life depends strictly on the economic factors of the individual patient.

As a rule, this device is indicated in patients aged ≥18 years, with moderate-severe OSA, BMI ≤35 kg/m^2^, AHI ≥15, intolerant to CPAP for ≥ 3 months, and without concentric collapse of the soft palate. In Spain, it has been evaluated [34], but no decision about its inclusion in the national health system has been made. However, in selected patients with moderate to severe OSA who do not tolerate or do not accept CPAP, the implant is used and financed in various European countries, such as England [35], France [36], the Netherlands [37], and in the USA [38] with F.D.A. approval [39].

Our study has some limitations. First: the number of patients implanted is small because the Spanish Public Health care system does not finance the device. Therefore, the patient must bear the procedure and device cost. To minimize this aspect, the study included all patients that were operated on in our center. Second: the study is retrospective, making it impossible to provide data that were not recorded at the initial time but could have been helpful. To minimize the loss of values, the follow-up time was reduced to 3 months. Third: given the small number of patients operated with this device, a prolonged patient recruitment period was needed, which could potentially bias the study based on possible variations in clinical practice. Fourth: all identified risk factors could not be included, as OSA is a multifactorial disease. Fifth: despite not being a control matched study, the baseline characteristics of the two groups excluded significant differences between them.

Despite the great difficulty in our country to implant patients with Inspire^®^, there is a need to carry out new randomized and prospective studies, which include more extensive size and a longer follow-up time, allowing a better analysis of the evolution of the IQoL.

## 5. Conclusions

The results indicate that, in patients with moderate to severe OSA who do not tolerate or do not accept standard treatment with CPAP, the HNS, through the implantation of the Inspire^®^ device, improves the patient’s quality of life to values equivalent to those of the general Spanish population. As it is not included in the portfolio of benefits of the Spanish health system, patients who do not have access to the device (mainly due to economic reasons) continue to have a reduced quality of life.

## Figures and Tables

**Table 1:** Baseline characteristics of the patients.

Variable	Total (*n* = 66)	IGr (*n* = 22)	CGr (*n* = 44)	*p*
Sex, male (%)	84.8 (4.4)	90.9 (6.1)	81.8 (5.8)	0.476
Age (years)	53.5 (13.0)	51.7 (11.2)	54.0 (13,9)	0.490
BMI (kg/m^2^)	28.7 (4,6)	28.1 (3.7)	29.05 (5.0)	0.456
AHI (events/hour)	39.7 (20.1)	42.9 (21.1)	43.7 (24.7)	0.928
ESS (SD)	11 (5.3)	12.1 (5.1)	10.4 (5.4)	0.227
HTN, % (SD)	40.9 (6.1)	50.0 (10.7)	36.4 (7.3)	0.304
DM, % (SD)	21.2 (5.0)	18.2 (8.2)	22.7 (6.3)	0.759
MI, % (SD)	9.1 (3.5)	9.1 (6.1)	9.1 (4.3)	1.000
Asthma, % (SD)	21.2 (5.0)	27.3 (9.5)	18.2 (5.8)	0.524
COPD, % (SD)	6.1 (2.9)	9.1 (6.1)	4.5 (3.1)	0.596
CRF, % (SD)	6.1 (2.9)	4.5 (4.4)	6.8 (3.8)	1.000
Dyslipidemia, % (SD)	47.0 (6.1)	50.0 (10.7)	45.5 (7.5)	0.797
Cognitive failure, % (SD)	4.5 (2.6)	4.5 (4.4)	4.5 (3.1)	1.000
Chronic pain, % (SD)	18.2 (4.7)	31.8 (9.9)	11.4 (4.8)	0.086
RLS, % (D SD E)	21.2 (5.0)	22.7 (8.9)	20.5 (6.1)	1.000
CPAP previous, % (SD)	78.8 (5.06)	86.4 (7.3)	75.0 (6.5)	0.354
Daily use de CPAP, % (SD)	21.2 (5.0)	13.6 (7.3)	25.0 (6.5)	0.354

BMI: body mass index; AHI: apnea–hypopnea index; ESS: Epworth sleepiness scale; HNT: arterial hypertension; DM: mellitus diabetes; MI: Myocardial Infarction; COPD: chronic obstructive pulmonary disease; CRF: chronic renal failure; RLS: restless legs syndrome; CPAP: continuous positive airway pressure. n: number of patients; SD: standard deviation.

**Table 2:** Patient EQ-5D-5D Utility Index Values.

	Unadjusted		Adjusted	
Group	Mean	SD	Mean	SD
IGr-pre	0.764	0.190	0.750	0.061
CGr-pre	0.733	0.205	0.763	0.060
IGr-post	0.935	0.101	0.814	0.056
CGr-post	0.727	0.200	0.765	0.063

SD: standard deviation; IGr-pre: Intervention group a t = 0 months; CGr-pre: Control group a t = 0 months; IGr-post: Intervention group a t = 3 months; CGr-post: Control group a t = 3 months.

**Table 3:** Percentage of patients without any problem in each dimension.

Dimension	Intervention, %; (SD)	Control, % (SD)	Difference, % (SD)	*p*
Mobility	95.5 (21.3)	68.2 (47.1)	27.3 (8.4)	0.002 *
Self-care	100.0 (0)	77.3 (42.4)	22.7 (6.4)	0.001 *
Usual activities	81.8 (39.5)	22.7 (42.4)	59.1 (10.6)	0.000 *
Pain/discomfort	72.7 (45.6)	43.2 (50.1)	29.5 (12.3)	0.020 *
Anxiety/Depression	77.3 (42.9)	22.7 (42.9)	54.5 (11.2)	0.000 *

SD: standard deviation; *: significant difference.

**Table 4:** Comparison of the usefulness index at 3 months of the study groups related to the Spanish population.

Utility Index	Difference with Spanish Population: Mean (95% CI)	*p*
Intervention	0.012 (−0.03 to 0.057)	0.578 **
Control	−0.196 (−0.257 to −0.135)	<0.001 *

95% CI: 95% confidence interval; *: significant difference; **: no significant difference.

**Table 5:** Comparison of the percentage of patients without problems in each dimension of quality of life at t = 3 months of the study groups related to the Spanish population.

Variable	Spanish Population (*n* = 66) %	Difference with IGr (*n* = 22) % (95% CI)	Difference with CGr (*n* = 44) % (95% CI)
Mobility	91.9	3.5 (−5.9 to 13.0) **	−23.7 (−38.1 to −9.4) *
Self-care	97.8	2.2 (n.a.)	−20.5 (−33.4 to −7.6) *
Usual activities	94.8	−13.0 (−30.5 to 4.5) **	−72.1 (−85.0 to −59.2) *
Pain/discomfort	81.9	−9.1 (−29.3 to 11.1) **	−38.7 (−53.9 to −23.4) *
Anxiety/Depression	86.4	−9.1 (−28.2 to 9.9) **	−63.7 (−76.6 to −50.8) *

95% CI: 95% confidence interval; IGr: Intervention group; CGr: Control group; n.a. not applicable; *: significant difference; **: no significant difference.

## Data Availability

The data underlying this article will be shared on reasonable request to the corresponding author.
